# Reinfection rate in a cohort of healthcare workers over 2 years of the COVID-19 pandemic

**DOI:** 10.1038/s41598-022-25908-6

**Published:** 2023-01-13

**Authors:** Ana Rubia Guedes, Maura S. Oliveira, Bruno M. Tavares, Alessandra Luna-Muschi, Carolina dos Santos Lazari, Amanda C. Montal, Elizabeth de Faria, Fernando Liebhart Maia, Antonio dos Santos Barboza, Mariana Deckers Leme, Francis M. Tomazini, Silvia Figueiredo Costa, Anna S. Levin

**Affiliations:** 1 Infection Control Department, Hospital das Clinicas, Faculdade de Medicina, Universidade de São Paulo, São Paulo, Brazil; 2 Instituto de Medicina Tropical, Faculdade de Medicina, Universidade de São Paulo, Av. Dr. Enéas Carvalho de Aguiar, 470, São Paulo, 05403-000 Brazil; 3 Department of Infectious Diseases, Faculdade de Medicina, Universidade de São Paulo, São Paulo, SP Brazil; 4 Central Laboratory Division, Hospital das Clinicas, Faculdade de Medicina, Universidade de São Paulo, São Paulo, Brazil; 5 Central Administration of Hospital das Clinicas, Faculdade de Medicina, Universidade de São Paulo, São Paulo, Brazil; 6 Healthcare Worker Health Services, Hospital das Clinicas, Faculdade de Medicina, Universidade de São Paulo, São Paulo, Brazil

**Keywords:** Viral infection, Infectious-disease diagnostics

## Abstract

In this large cohort of healthcare workers, we aimed to estimate the rate of reinfections by SARS-CoV-2 over 2 years of the COVID-19 pandemic. We investigated the proportion of reinfections among all the cases of SARS-CoV-2 infection from March 10, 2020 until March 10, 2022. Reinfection was defined as the appearance of new symptoms that on medical evaluation were suggestive of COVID-19 and confirmed by a positive RT-PCR. Symptoms had to occur more than 90 days after the previous infection. These 2 years were divided into time periods based on the different variants of concern (VOC) in the city of São Paulo. There were 37,729 medical consultations due to COVID-19 at the hospital’s Health Workers Services; and 25,750 RT-PCR assays were performed, of which 23% (n = 5865) were positive. Reinfection by SARS-CoV-2 was identified in 5% (n = 284) of symptomatic cases. Most cases of reinfection occurred during the Omicron period (n = 251; 88%), representing a significant increase on the SARS-CoV-2 reinfection rate before and during the Omicron variant period (0.8% vs. 4.3%; *p* < 0.001). The mean interval between SARS-CoV-2 infections was 429 days (ranged from 122 to 674). The Omicron variant spread faster than Gamma and Delta variant. All SARS-CoV-2 reinfections were mild cases.

## Introduction

After over 2 years of COVID-19 pandemic, reinfections are still a debated topic^1^. Reinfection by SARS-CoV-2 means that a person was infected, recovered, and became infected again at least 90 days after the last infection^2^. Most individuals will develop some protection against reinfection after recovering from COVID-19; however, the evidence regarding duration and level of protection is still emerging^1^. Reinfections have been described although less severe and less lethal than primary infections^3^. The frequency with which reinfections occur, how quickly occur after a previous infection, and the severity in relation to initial infections are relevant information to understand the phenomenon and the impact of reinfections^2^. Reinfection cases were rare during the first year of pandemic^4^. However, a study coordinated by the Centers for Disease Control and Prevention (CDC) found an increase in reinfection cases during the Omicron period represented by 50% of reinfections^5^. Immunity acquired due to an infection by one variant of the virus may not be effective against a new variant^6^, and immunity acquired by vaccination may offer varying protection against different SARS-CoV-2 variants^3^.

The objective of this study is to estimate the rate of reinfections with SARS-CoV-2 in healthcare workers over 2 years of the COVID-19 pandemic.

## Results

From March 10, 2020 until March 10, 2022 there were 37,729 medical consultations due to COVID-19 at the hospital’s Health Workers Services; 25,750 RT-PCR tests were done for suspected COVID-19 of which 23% (n = 5865) were positive. A total of 284 (5%) cases were considered SARS-CoV-2 reinfections. These cases belonged to 281 HW, of which three of them had a second episode of reinfection during the Omicron variant period. Our cohort was predominantly female (n = 202/281, 71%) with a median age of 39 years (30–47), 4% were unvaccinated, 32% had received two doses of a COVID-19 vaccine and 64% a booster dose. In addition, 22% (n = 62/281) had at least one risk factor for progression to severe COVID-19, mainly cardiovascular disease, diabetes, and older age. However, all cases were mild, there were no hospitalizations or deaths (Table 1).

**Table 1:** Characteristics of 281 healthcare workers (HW) of Hospital das Clinicas (University of São Paulo) with reinfection by SARS-CoV-2 during 2 years of the pandemic (March 10, 2020–March 10, 2022).

Characteristics	N (%) or median (IQR)
**Female**	202 (71)
**Age**	39 (30–47)
**Number of HW with risk factors**	62 (22)
Cardiovascular disease (including hypertension)	42 (15)
Diabetes	15 (5)
≥ 60 years	9 (3)
Chronic respiratory disease	7 (2)
Malignancy	6 (2)
Obesity	5 (2)
Pregnancy	1 (0)
**Number of risk factors per HW**	
1	48 (17)
≥ 2	11 (4)
**Mild COVID-19**	281 (100)
**Vaccination status**	
Unvaccinated	10 (4)
Vaccinated with two doses	90 (32)
Vaccinated with a booster dose	181 (64)

Table 2 displays a distribution of COVID-19 cases according to the emergence of VOCs in the city. Most cases of reinfection occurred during the Omicron VOC period (n = 251; 88%). The mean interval between infections was 429 (122–674) days (approximately 14 months); and was 507 (122–674) days (approximately 16 months) in the Omicron period.

**Table 2:** Distribution of COVID-19 cases in healthcare workers (HW) of Hospital das Clínicas (University of São Paulo) according to the emergence of variants of concern (VOCs) during 2 years of the pandemic (March 10, 2020-March 10, 2022).

Variables	Pre-VOC period	Transition to Gamma*	Gamma period	Transition Gamma to Delta	Delta period	Transition Delta to Omicron	Omicron period
	10 March–30 November, 2020	1 December, 2020–14 March, 2021	15 March–2 August, 2021	3 August–16 September, 2021	17 September–2 December, 2021	3–31 December, 2021	1 January–10 March, 2022
Duration of the period (days)	265	103	140	44	76	28	68
Number of HW tested	10,195	3063	4732	1679	2101	894	3086
Positives for SARS-CoV-2	2803	602	476	163	103	52	1666
Positivity rate (n of positives/n of HW tested)	27%	20%	10%	10%	5%	6%	54%
Mean number of new cases per day	10.6	5.8	3.4	3.7	1.4	1.9	24.5
**Number of cases of reinfection**	**0**	**7**	**10**	**6**	**4**	**6**	**251**
Period in which primary infections occurred	**–**	Pre-VOC = 7	Pre-VOC = 9 Transition to Gamma = 1	Pre-VOC = 5 Transition to Gamma = 1	Pre-VOC = 4	Pre-VOC = 5 Transition to Gamma = 1	Pre-VOC = 185 Transition to Gamma = 34 Gamma period = 25 Transition Gamma to Delta = 3 Delta period = 1
**Reinfection rate** (number of reinfections/total cases)	–	**0.2%**	**0.3%**	**0.2%**	**0.1%**	**0.1%**	**4.2%**
Mean interval between infections in days (range)	–	258 (193–316)	344 (126–434)	359 (187–486)	533 (506–562)	505 (319–605)	507 (122–674)
Reinfection in vaccinated HW (%)	–	2 (29)	10 (100)	5 (83)	4 (100)	6 (100)	244 (98)
Reinfection in vaccinated with additional dose (%)	–	NA	NA	NA	2 (50)	2 (33)	177 (71)
Number of occurrences of a 3rd infection	–	0	0	0	0	0	3

Most important values are in bold.

*NA* not applicable.

*Vaccination campaign against COVID-19 in HC.

There was a significant increase on the SARS-CoV-2 reinfection rate comparing the number of cases among before and during the Omicron variant period (0.8% vs. 4.3%; relative risk 5.45 [95% IC 3.80–7.81]; *p* < 0.001).

At the same time, the average number of new cases in our HW also increased drastically with an average of 24.5/day compared with 10.6/day in 2020 (before vaccination).

## Discussion

HWs are considered to be at high risk of infection as they face the possibility of HW-to-HW transmission, patient-to-HW transmission, and community transmission^7^. Before the predominance of the Omicron variant in the city of Sao Paulo, reinfection represented less than 1% of symptomatic COVID-19 cases similar to previously described^8,9^. Moreover, higher reinfection rates have been described varying from 3.2 to 11.6% including asymptomatic cases^10,11^. However, in the Omicron period, this proportion increased more than five times. The Omicron variant spread faster than Gamma and Delta variants, evidenced by the progressively shorter duration of the transition periods. Nevertheless, during the Gamma and Delta periods, reinfection was rare, suggesting that previous infection and vaccination had an impact. This effect seems to be less important against the Omicron variant.

SARS-CoV-2 reinfections by natural immunity can last over a year but declines progressively^3,12^. In addition, it has been described that protection against reinfection varies between 77 and 98% across different SARS-CoV-2 variants, but few data are available regarding the Omicron variant^3^.

Furthermore, considering the long interval between the infections, it is possible that immunity, acquired through infection and vaccination, waned over time as previously shown^3,12^. Our data reinforced that the median interval between infections is approximately 14 months similar to previous studies^3,12^.

An important finding was that all reinfections were non-severe. However, most HW do not belong to high-risk groups and are mostly younger than 65. Another finding was that, in most cases of reinfection, the first infection occurred in the pre-VOC period as previously described^3^.

Our study has limitations. This is a single-center study; however, our number of tests was high. It was only possible to evaluate the number of tests performed at our hospital. We believe that most HW chose to test at our service, as testing was conveniently done at the workplace and free of charge. Furthermore, as HW were vaccinated during the evaluation period, it was not possible to evaluate separately the impact of infection and vaccination. We did not determine variants of concern by SARS-CoV-2 whole genome sequencing. Finally, only symptomatic infections were evaluated.

In summary, in our large 2-year cohort of HW, reinfection represented 5% of symptomatic COVID-19 cases. All reinfections were mild and occurred predominantly in the Omicron period.

## Methods

This is a prospective cohort study of HW with suspect COVID-19 symptoms. We evaluated all the HW who were diagnosed with COVID-19 with a positive RT-PCR test between March 10, 2020 and March 10, 2022. Demographic and clinical data were obtained from electronic health records.

Hospital das Clínicas (HC) is a 2200-bed tertiary care hospital affiliated with the University of São Paulo, Brazil, located in the city of São Paulo (> 12 million inhabitants)^13^. It has approximately 22,000 directly hired healthcare workers (HW), which who provide direct patient assistance and administration.

During the pandemic, symptomatic HW were evaluated by a physician at the hospital’s Health Worker Services and tested for SARS-CoV-2 by RT-PCR^14^ in accordance with relevant guidelines and regulations. HW received paid leave during symptomatic illness, and if positive for SARS-CoV-2 their leave was extended until the 10th day since the onset of symptoms. No further testing was required for returning to work.

The vaccination campaign with two doses of inactivated SARS-CoV-2 vaccine (CoronaVac/Sinovac, China) began in January, 2021. During the first months, 22,402 and 21,652 HWs have received the first dose and 2nd dose, respectively. In October, 2021 the government issued a recommendation that HWs take a 3rd dose of BNT162b2 (Pfizer-Biontech, USA). This was not organized and offered by the hospital and there are no data on adherence.

The 2 years of pandemic were divided into time periods based on the different variants of concern (VOC) in the city of São Paulo:

1. *Pre-VOC period* from the first reported case of COVID-19 until the day before the first case report of the Gamma variant,
2. *Transition to Gamma* from the day of the first case reported of the Gamma variant until the day before the report that 90% of cases were caused by the Gamma variant,
3. *Gamma period* from the day of the report that 90% of cases were caused by the Gamma variant until the day before the first case report of the Delta variant,
4. *Transition Gamma–Delta* from the day of the report of the first case of the Delta variant until the day before the report that 90% of cases were caused by the Delta variant,
5. *Delta period* from the day of the report that 90% of cases were caused by the Delta variant until the day before the report of the first case of the Omicron variant,
6. *Transition Delta–Omicron* from the day of the first case report of the Omicron variant until the day before the report that 90% of cases were caused by the Omicron variant,
7. *Omicron period* from the day of the report that 90% of cases were caused by the Omicron variant until the end of the study period.

The main outcome was the proportion of reinfections among all the cases of COVID-19 in each period.

Reinfection was defined as the appearance of new symptoms that on medical evaluation were suggestive of COVID-19, confirmed by a positive RT-PCR test for SARS-CoV-2. Symptoms had to occur more than 90 days after the previous diagnosis of a RT-PCR-confirmed infection.

A mild COVID-19 case was considered as a HW who presented at least one of the following symptoms: fever or chills, cough, runny nose, sore throat, headache, nauseas or vomiting, myalgia, fatigue, diarrhea, anosmia, and ageusia. Moderate cases were defined as symptomatic cases who have shortness of breath, dyspnea, or an abnormal chest imaging with an oxygen saturation (SpO_2_) ≥ 94% on room air at sea level. Severe cases presented a SpO_2_ < 94%, respiratory rate > 30 breaths/min or a pulmonary infiltrate > 50% and required hospitalization. Finally, critical illness was considered as HW with respiratory failure, septic shock and/or multiple organ dysfunction^15^.

This study was analyzed and approved by the Ethics committee of the Hospital das Clínicas of the Faculty of Medicine of the University of São Paulo with a waiver of informed consent (CAAE number: 60253222.5.0000.0068).

### SARS-CoV-2 RT-qPCR

RNA was extracted from saline solution 0.9% with an automated method using magnetic beads (Sample Preparation System RNA, Abbott, Illinois, USA). SARS-CoV-2 RT-qPCR was performed using an adapted protocol as previously described^8^. Gene E was detected as the first-line screening tool, followed by confirmatory testing with an assay detecting the N gene (Abbott, USA) and the commercial SARS-CoV-2 N1 + N2 RT-qPCR kit to detect N1 and N2 genes (Qiagen, USA). SARS-CoV-2 RT-qPCR result was considered positive with an amplification cycle threshold (Ct) ≤ 32 and Ct ≤ 33, respectively.

### Data analysis

Demographic and clinical characteristics were presented as frequencies (percentages) for categorical variables and means (range) or medians (interquartile range [IQR]) for continuous variables according to the type of distribution. The comparison of SARS-CoV-2 reinfection rates was performed using the Chi square test (*p* < 0.05, 95% confidence intervals) and were calculated as number of reinfection cases before and after the Omicron variant considering the total accumulated number of SARS-CoV-2 infections in both periods. Epi Info™ version 7.2.4.0 was used for statistical analyses.

## Data Availability

The datasets used and/or analyzed during the current study are available from the corresponding author on reasonable request.
